# Sexual dimorphism in chronic respiratory diseases

**DOI:** 10.1186/s13578-023-00998-5

**Published:** 2023-03-07

**Authors:** Karosham Diren Reddy, Brian Gregory George Oliver

**Affiliations:** 1 Respiratory and Cellular Molecular Biology Group, Woolcock Institute of Medical Research, Glebe, NSW 2037 Australia; 2 School of Life Science, University of Technology Sydney, Ultimo, NSW 2007 Australia

**Keywords:** Sexual dimorphism, Asthma, COPD, Lung cancer, Sex chromosomes, Inflammation, Remodelling

## Abstract

Sex differences in susceptibility, severity, and progression are prevalent for various diseases in multiple organ systems. This phenomenon is particularly apparent in respiratory diseases. Asthma demonstrates an age-dependent pattern of sexual dimorphism. However, marked differences between males and females exist in other pervasive conditions such as chronic obstructive pulmonary disease (COPD) and lung cancer. The sex hormones estrogen and testosterone are commonly considered the primary factors causing sexual dimorphism in disease. However, how they contribute to differences in disease onset between males and females remains undefined. The sex chromosomes are an under-investigated fundamental form of sexual dimorphism. Recent studies highlight key X and Y-chromosome-linked genes that regulate vital cell processes and can contribute to disease-relevant mechanisms. This review summarises patterns of sex differences in asthma, COPD and lung cancer, highlighting physiological mechanisms causing the observed dimorphism. We also describe the role of the sex hormones and present candidate genes on the sex chromosomes as potential factors contributing to sexual dimorphism in disease.

## Background

Sexual dimorphism refers to a divergence in the physical characteristics between chromosomally defined males and females of a species. These differences exist at the organ, cellular and molecular levels and are critical for establishing differences between males and females and enabling sexual reproduction [1–3]. However, the potential for these differences to contribute to sex differences in susceptibility and disease development is overlooked. Recently, the scientific community has actively aimed to recognise and investigate trends of sex differences both epidemiologically and physiologically. For example, the National Institutes of Health (NIH) mandated that sex must be considered a critical biological variable [4]. This instruction highlights the lack of data investigating sex as a biological factor in disease development and progression. The NIH highlights that sex is a biological variable that should be considered at all levels of research, from experimental design to analysis and reporting findings in animal and human studies.

It is critical to define the difference between biological sex and gender. Biological sex refers to the sex chromosome complement of an individual. Males carry one Y-chromosome, and one X-chromosome (XY), whilst females have two X-chromosomes (XX). The presence of the *SRY* gene on the Y-chromosome initiates a hormone cascade during early development, stimulating the formation of the characteristic male phenotype. In contrast, the absence of the *SRY* gene results in the generation of female characteristics. Gender is defined by social norms and expectations for how “men and women” should behave [5]. The factors that influence gender vary between different cultures and with time. Notably, a growing body of work recognises the complex interaction between gender and disease outcomes. Although important, the impact of gender on disease is beyond the scope of the current body of work, and biological sex differences between males and females will be the primary focus of this review.

The reporting and investigation of sex differences in disease are being increasingly recognised across various health conditions [6]. Nonetheless, there remains an incomplete understanding of the molecular and genetic factors driving sexual dimorphism. This is partly a result of large clinical and cohort studies designating sex as a confounding factor or a covariate in the data analyses [7]. As a result, the complexities of diseases remain poorly understood or unidentified as sometimes the effects of disease between males and females may occur in opposing directions, resulting in a “net-zero” effect size when grouped [8]. When public RNA-seq datasets are stratified by sex, significant differences in gene expression are apparent between males and females in non-gonadal tissues, which are otherwise non-significant when unstratified [8, 9]. As a result, a considerable gap exists in our understanding of the fundamental differences between males and females. Sex differences in response to the same clinical interventions are well reported in the literature to affect patient outcomes [7, 10–12]. Developing a deeper understanding of the fundamental factors and mechanisms driving sexual dimorphism in diseases is critical to furthering our understanding of disease development and creating new, more effective ‘personalised’ clinical treatments.

Here, we will review patterns of sex differences in prominent respiratory diseases and present how sexual dimorphisms manifests at a molecular and physiological level. We will also explore how the sex hormones and sex chromosomes contribute to pathological differences between males and females.

### Sexual dimorphism in lung physiology

Differences in the lung structure between males and females may contribute to patterns of sexual dimorphism in various respiratory diseases. The lung’s development and maturation present a complex and dynamic pattern of sexual dimorphism driven by various factors. Importantly, differences in lung physiology between males and females have important clinical implications. Male lungs are bigger than female lungs, with this difference existing from birth into adulthood [13]. The disparity in lung development in utero between male and female foetuses begins as early as 16 to 24 weeks gestation [14]. Female foetuses have smaller airways and a lower number of respiratory bronchioles compared to males; however, their maturation rate is faster. Surfactant, an essential compound enabling correct lung function [15], is produced earlier in females than males, enabling a faster lung maturation rate. The faster rate of development is thought to explain why female neonates are less likely to suffer from respiratory distress syndrome compared to male neonates [16]. Estrogen produced by the placenta stimulates the production of surfactant and the development of alveoli [17]. In contrast, testicular-derived androgens such as testosterone function to suppress the production of surfactant [13, 17], to which female foetuses are not exposed. As a result of divergent patterns of lung development, in early life, males and females present with distinct physiological lung profiles. As mentioned above, female lungs are smaller, with fewer respiratory bronchioles and smaller airways [14], whilst the luminal area for the large and central airways is approximately 14–31% larger in males, even when matching for lung size [18]. Cumulatively, as the female lung is smaller, with fewer respiratory bronchioles, the total number of alveoli and lung surface area is higher for males throughout early development. This disproportionate lung size and airway growth rate is called ‘dysanapsis’ [19, 20]. Females demonstrate higher forced expiratory flow rates until they are 18 years old [21]. This increased airflow rate is postulated to reduce female children’s susceptibility to damage due to in utero exposures and the development of respiratory conditions such as asthma and respiratory tract infections [6, 22]. As total lung capacity (TLC) increases in females, sex differences in expiratory flow rates also diminish. Clinical studies attribute sex differences in lung pathology changes such as airway fibrosis and inflammation to physiological and anatomical differences [23]. As such, biological and structural differences between males and females may contribute to patterns of sexual dimorphism in respiratory diseases such as asthma, chronic obstructive pulmonary disease (COPD) and lung cancer.

### Sex differences in respiratory diseases

As mentioned, sexual dimorphism is apparent in a range of diseases across multiple organ systems. However, sex differences in the susceptibility, severity and progression between males and females for chronic respiratory diseases are particularly intricate. For example, idiopathic pulmonary fibrosis is two times more common in males [24], whilst cystic fibrosis demonstrates greater severity in women [25]. This complex interaction between sex and disease becomes significantly apparent for asthma, chronic obstructive pulmonary disease (COPD) and lung cancer.

#### Asthma

**Fig. 1:**
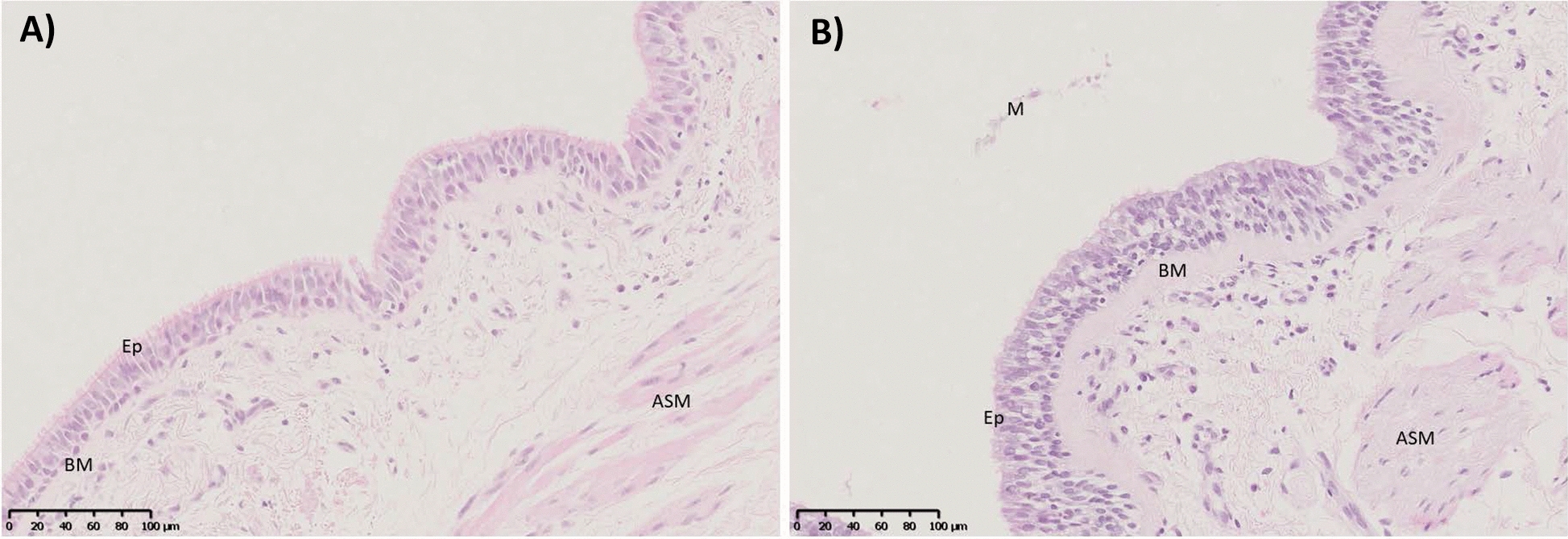
Micrographs of airways from a healthy patient **A** and an asthmatic patient **B** stained with haematoxylin and eosin. A thickened basement membrane (BM) can be seen in the asthmatic patient with hyperplasia of the epithelial (Ep) layer. A noticeable increase in the airway smooth muscle (ASM) thickness can also be seen. A slight mucus exudate can be seen in the airway lumen of the asthma patient. Scale bars = 100 µm

Asthma is a heterogenous respiratory disease characterised by hyper-reactive and reversible airway inflammation. It is primarily diagnosed based on a history of respiratory symptoms from wheezing, episodic shortness of breath (dyspnea), chest tightness and cough, varying over time [26]. Intrinsic to asthma is a complex interplay between airway inflammation and remodelling, culminating in airway hyperresponsiveness (AHR). Airway remodelling refers to structural changes in the airways, such as increased airway smooth muscle (ASM), thickened basement membrane, epithelial dysplasia, and increased collagen deposition [27]. These changes result in a thickened airway wall, which, combined with inflammatory exudate produced by immune cells, obstruct the airway causing difficulty breathing. Figure 1 illustrates the significant obstruction of the airway lumen in asthma patients compared to healthy patients. The extent of expiratory airflow limitation is measured by the forced expiratory volume in one second (FEV_1_), which is a common tool used to evaluate asthma. Variability in FEV_1_ is commonly triggered by exposures such as exercise, allergens or viral infections. In most cases, as asthma becomes more severe the airflow obstruction becomes fixed, increasing the rate of FEV_1_ loss.

The exact cause of asthma remains unknown. As such, it remains a significant health problem. Approximately 300 million people suffer asthma worldwide, with Australia demonstrating one of the highest prevalence rates, at 11.2% [28, 29]. Asthma is the leading disease burden for children younger than 15, with children who are under 15 more likely to be hospitalised with asthma than those older than 15 [30]. Part of the difficulty in treating and managing asthma relates to its heterogeneous nature, with five primary clinical phenotypes recognised. A range of clinical patterns or treatment responses determines the different asthma phenotypes. These include the causative agents (allergic), timing of diagnosis (adult-onset), lung function outcome (persistent airflow limitation) and associated comorbidities [26]. Allergic asthma is the most common phenotype, often starting in childhood and driven by eosinophilic airway inflammation in response to stimulation by allergens. Non-allergic asthma, on the other hand, presents a more neutrophilic immune cell profile and responds less to inhaled corticosteroids (ICS). Significant advances have been made regarding asthma management and treatment; however, the exact cause of asthma remains elusive. Furthermore, modern treatments are flawed, with therapies such as targeted monoclonal antibodies failing to eliminate dangerous exacerbations [31]. Other clinical interventions only target specific aspects of asthma, such as anti-inflammatory steroids that reduce the immune response but do not resolve the structural changes that occur [31]. This inability to resolve airway remodelling limits the ability to control patients with severe disease and presents a significant shortfall in the attempt to cure/reverse asthma.

#### Sex differences in asthma

**Fig. 2:**
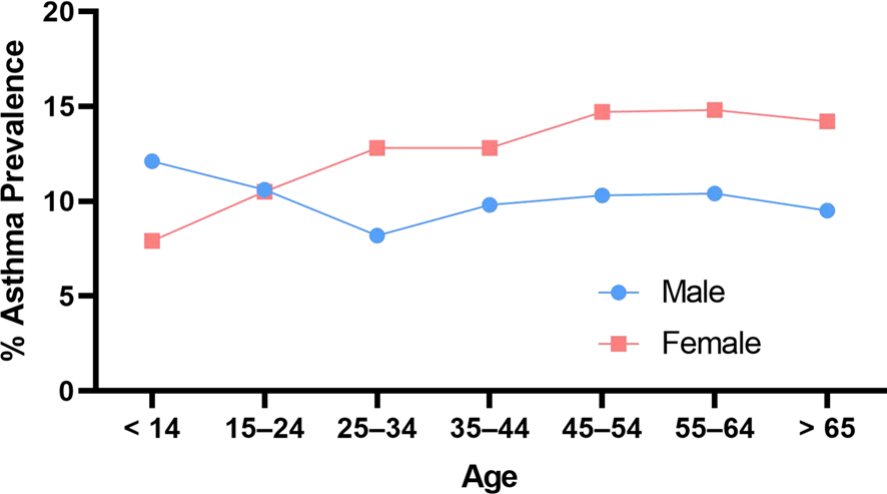
Asthma prevalence by age group and sex in Australia recorded by the National Health Survey 2017–2018 [29]. The pink line indicates female asthma prevalence (%), and the blue line indicates male asthma prevalence (%)

Compounding the complexity of asthma is its sexually dimorphic nature. Differences between males and females with asthma exist in childhood and adulthood asthma. Young males (12.1%) report a higher prevalence of asthma compared to young females (7.9%) and are two times more likely to be hospitalised due to asthma [32]. However, this pattern reverses after puberty, towards an increased asthma diagnosis in adult females (13.9%) compared to adult males (9.6%), as depicted in Fig. 2. However, some conjecture exists in the literature, with some studies concluding no sex differences in asthma severity [33, 34]. Other studies report higher mortality and a higher rate of exacerbations in females [23], severely reducing their daily quality of life. In contrast, adult females have a three-fold increased risk for hospitalisation due to asthma [32]. Several factors contribute to the sexual dimorphic presentation of asthma, including anatomical differences, sex hormones and environmental/occupational factors.

The apparent differences in asthma susceptibility and severity between the sexes are associated with many factors. The differential inflammatory, fibrotic, and remodelling processes present a complex nexus of physiological and molecular factors contributing to asthma pathogenesis and progression. The sex hormones have been linked with the temporal shift in asthma susceptibility [35–37]. However, genome studies reveal genetic associations in asthma differ by sex, highlighting unique biological underlying factors contributing to sexual dimorphism in asthma [8]. Network analyses conclude that asthma may function via differential mechanisms in males or females, despite involving similar processes and functional outcomes [38]. Therefore, a holistic understanding of environmental, genetic, hormonal and physiological factors is needed to unravel the complex interaction between asthma and biological sex.

#### Sex differences in inflammation and remodelling in asthma

A predominance of CD4 + and Th2 cells with infiltration of eosinophils and mast cells in the airways characterises type 2 airway inflammation. CD4 + and T helper cells initiate and perpetuate the phenotype of prolonged inflammation [39]. This type of inflammation primarily drives allergic/atopic asthma. In particular, it has been noted that young males present with more allergic inflammation [40]. Interestingly, males are less prone to immunological illnesses over their lifetime than females [23]. This pattern has been linked to the role of sex hormones, which will be discussed in detail later. Males and females demonstrate distinct immune cell populations in asthma. Female lungs have increased levels of type 2 lymphoid cells, specifically a subset of cells that do not express killer-cell lectin-like receptor G1—which is absent from male lungs [41]. After puberty, this cell population can produce type 2 inflammatory cytokines, thus creating different pro-inflammatory environments between males and females with asthma. There is a distinct pattern towards more atopic asthma, airway infections and bronchiolitis in young males before puberty, with more males admitted to hospital before puberty [39]. The differences between male and female immunological mechanisms and responses are complex and change with age.

Tumour necrosis factor—alpha (TNFα) and transformation growth factor—beta (TGFβ) are prominent immunoregulatory cytokines closely associated with asthma pathogenesis [42]. They are critical to asthma's cellular and humoral immune responses [43]. Associations have been identified between genetic polymorphisms in atopic and non-atopic asthma patients [44, 45]. In particular, they have been correlated with serum IgE levels, of which boys demonstrate higher levels than girls [28]. However, the allergic response to asthma allergens can depend on CD4 + and CD8 + cells rather than IgE levels [23]. As such, the CD4 + to CD8 + cell ratio can be a marker of chronic lung disease. This ratio is lower in males than females in adulthood [46], contributing to sex differences in the inflammatory response. TNFα demonstrates various pathological functions, including inducing the infiltration and activation of immune cells through promoting increased expression of adhesion molecules [47], thus increasing bronchial hyperresponsiveness. Further, TNFα dysregulates epithelial barrier activities along with IL-13 [48]. Female-biased expression of IL-13 in asthma patients may interrupt tight junction proteins, contributing to worse asthma symptoms in females. The IL-17 pathway is also up-regulated in females with asthma, potentially driving increased airway hyperresponsiveness [49]. Type 2 immune response in asthma increases the number of neutrophils driven by CXCL8, a well-known neutrophil chemotactic cytokine [23, 50]. Similarly, IL6 levels correlate with worse asthma outcomes as part of type 2 immune response causing neutrophil infiltration [51]. Neutrophilic asthma is linked with a poorer response to corticosteroids [10], impacting patient outcomes. Furthermore, asthmatic males demonstrate reduced response to β_2_-agonists with age despite treatment with inhaled steroids [27].

Airway remodelling refers to the degradation and repair of the ECM and the increased proliferation of fibroblasts. Traditionally it is believed that inflammation drives the airway remodelling in asthma, progressing into AHR and culminating in fixed airflow obstruction (FAO) [52]. However, there is a growing consensus that the altered structure of the airway may stimulate and promote inflammatory processes [53, 54]. For example, the breakdown of collagen IV affects asthma severity [45, 46] due to a decrease in the tumstatin fragment, reducing inflammation and AHR [55]. Therefore, sex differences in inflammation may drive sex differences in fibrosis and vice versa. Rasmussen et al. [27] found in a longitudinal population study that airway remodelling is associated with the male sex, with reduced lung function outcomes from childhood into adulthood. Males demonstrate an accelerated decline in FEV_1_ predicted values, potentially driven by higher rates of fixed airflow obstruction in younger and older populations [28, 56]. Despite this, female mice exposed to an Ova-sensitised model experience significantly more airway remodelling than male mice [57]. Lung function has been used as a surrogate method to measure the progression of airway remodelling and asthma, as an increased rate of FEV_1_ decline is seen in many asthma cases [52]. The ratio of FEV_1_ to vital capacity (the total volume of air that can be inhaled) indicates a downward trend in females from late adolescence into adulthood, signifying greater fibrotic and remodeling changes compared to males [27]. In early development, young males demonstrate slower expiratory airflow rates despite having similar total lung volumes to young females [39], indicating an initial structural disadvantage. Therefore, young males are more liable to develop asthma symptoms at a younger age, which might contribute to worse outcomes.

A complex relationship exists between inflammation, airway remodelling, biological sex and asthma. The exact mechanisms and factors causing these apparent differences in pathological processes remain unclear and require deeper investigation and discussion. Structural or functional sex differences are unlikely to drive respiratory disorders such as asthma and wheezing, with hormonal or genetic factors likely to contribute.

#### Chronic obstructive pulmonary disease (COPD)

COPD is characterised by progressive and irreversible airflow limitation, culminating in a sustained decline in lung function. Chronic inflammation of the airway drives the thickening and narrowing of the airway structural layers, obstructing airflow [58], similar to asthma. In contrast, COPD is characterised by small airway remodelling and emphysema, with occlusion of the airways and parenchymal destruction. COPD patients experience significant airflow limitation which is presented through the hallmark features of chronic cough, shortness of breath and excessive mucous production [59]. The irreversible worsening of disease symptoms significantly reduces patient quality of life, eventuating in disability and death. COPD is currently the third leading cause of death worldwide, affecting 7.5% of Australians older than 40 and 30% of people older than 75 [60]. Cigarette smoking is the best-known risk factor for the development of COPD; however, the exact cause of COPD remains unknown, with only 10% of COPD cases attributable to genetic factors. It is generally considered an adult-onset disease, and lifelong exposure to environmental factors functions as a critical pathogenic factor.

#### Sex differences in COPD

**Fig. 3:**
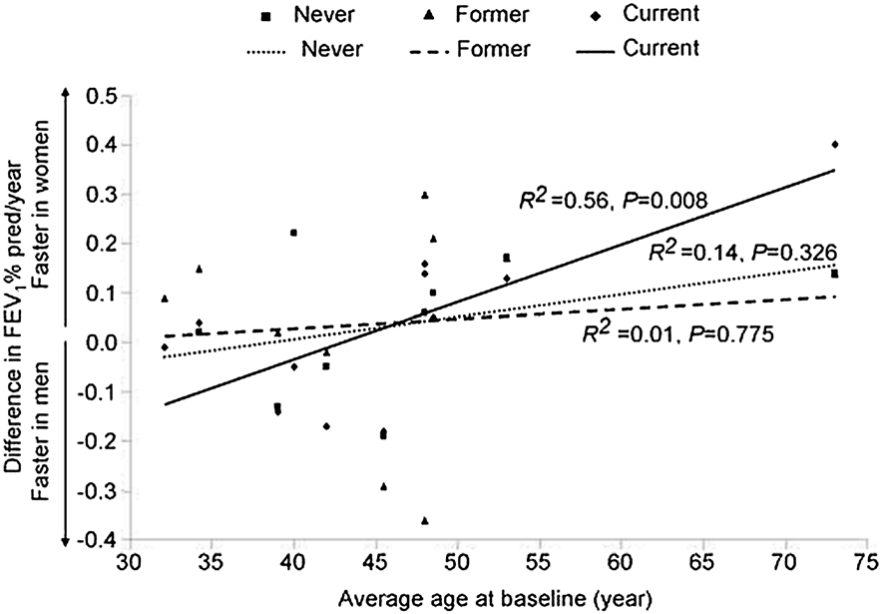
Unweighted analysis of the relationship between age and gender-related differences in the annual decline in lung function (FEV_1_%) pred/year according to smoking. The squares/dotted line represents never smokers, the triangles/dashed line represents former smokers, and the diamonds/solid line represents current smokers. *Recreated with permission from BioMed Central publisher and was first published by Gan *et al*. *[64]

Historically, COPD was considered male predominant. In recent years, there has been increased awareness and investigation of sex differences in COPD incidence and health outcomes. The National Centre for Health Services (NCHS) data shows that COPD death rates declined for males yet remained steady in females over time [61]. This trend is driven by a normalisation of smoking rates between males and females, which has narrowed COPD diagnosis rates between the sexes. Female smokers are 50% more likely to develop COPD than males [62]. Tam et al. found that 60% of all COPD hospitalisations occurred in females [63]. When considering the effect of smoking further, females demonstrate worse lung function and disease prognosis than males despite smoking at the same level [64] (Fig. 3). As such, due to environmental exposures, females present a steeper decline in lung functionality, contributing to increased rates of COPD diagnosis [65]. Females showed a 5.7% reduction in FEV_1_% predicted in the low smoke exposure group compared to males [66]. Women report more dyspnoea, chronic cough and lower overall quality of life scores. In addition, females also represent a more significant proportion of severe early-onset COPD patients (SEO-COPD) [67], defined by the development of COPD before 50 years of age with lung function less than the lower limit of normal [62, 68]. Importantly, sex differences in COPD manifest at a pathophysiological level, with males demonstrating higher levels of emphysema, whilst females show more significant small airway disease. A mouse model by Tam et al. found female mice exhibit increased levels of small airway remodelling and greater activation of TGFβ in the small airways after chronic cigarette exposure [63], which was not observed for males. A proteomic investigation of bronchoalveolar lavage (BAL) revealed an increase in macrophage autophagy in females who had developed COPD [69]. Macrophage autophagy is known to be a critical process in the pathophysiology of COPD [70]. An autoimmune profile has been reported in COPD [71]. As autoimmune diseases demonstrate a distinct female-bias, this provides support of a molecular basis towards increased susceptibility of females to develop COPD [72]. Forsslund et al. show female smokers with COPD present with increased CD8 T-cells expressing CCR5 compared to non-smoker females with COPD [73]. The authors highlight distinct T-cell profiles dependent on smoking status, demonstrating a correlation between Th1 inflammation with goblet cell density and BAL macrophages in female smokers with COPD. Comparatively, a correlation between Th2 inflammation and IgG serum concentration was reported in male smokers, with no effect of COPD observed. As such, clinical observations of sex differences stem from distinct cellular and biological interactions with cigarette smoking and COPD development.

#### Lung cancer

Lung cancer is one of the most common cancer types, with rates continuing to increase globally [74, 75]. Overall, there is a trend towards increased lung cancer cases in never-smoking individuals, although 80% of all lung cancer cases are attributable to a history of tobacco smoking [76]. Lung cancer is broadly classified into two subgroups: non-small cell lung cancer (NSCLC), which comprises 85% of cases, and small cell lung cancer (SCLC), which accounts for 15% of patients [77]. NSCLC includes specific subtypes such as squamous cell carcinoma, large cell carcinoma from epithelial cells that line the bronchus, and adenocarcinoma from the gland tissues [78]. SCLC is characterised by a rapid doubling time and is the most aggressive, reporting a 5 year survival rate of less than 7% [79]. Significant genetic diversity in lung cancer complicates the investigation and understanding of biological pathways involved in disease development and progression. Advancements in modern sequencing technologies have identified key oncogenic targets such as *KRAS, EGFR, BRAF* and *JAK2* [80]. However, the complexity of lung cancer is attributed to its lack of recurrent mutations that occur at a high frequency. This phenomenon impedes the ability to develop more effect treatments [81]. Improving our knowledge of the fundamental pathological features of lung cancer will enable the identification of key, targetable pathways and ultimately improve patient outcomes.

#### Sex differences in lung cancer

Sex bias in lung cancer is well established, with notable differences observed since 1996 [82]. Lung cancer is the second most diagnosed malignancy and the leading cause of cancer death worldwide [77]. Females demonstrate more adenocarcinoma and less squamous cell carcinoma than males [77]. This pattern was thought to relate to differences in smoking patterns between the sexes. However, 50% of women diagnosed with lung cancer are never smokers, compared to 20% of males [83]. Thun et al. found female never smokers of European, African American and Asian descent all showed increased lung cancer rates compared to their male counterparts [84]. The combination of these trends spurred the notion that female lung cancers have a distinct genetic and pathogenic profile compared to males.

**Fig. 4:**
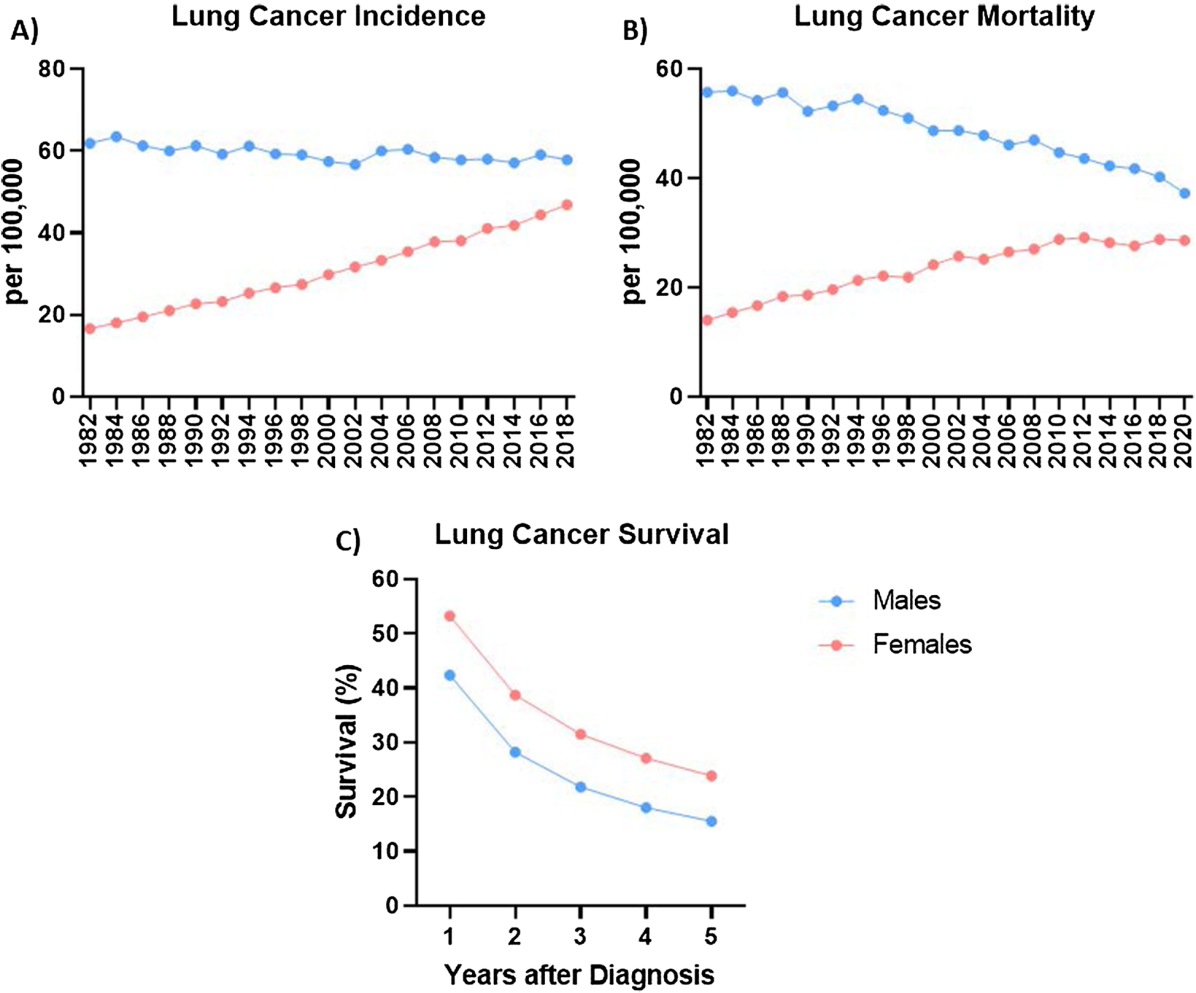
Lung cancer incidence, mortality and survival, collected by the Australian Institute of Health and Welfare [90]. **A** Age-adjusted lung cancer incidence rates by sex **B** age-adjusted lung cancer mortality by sex **C** lung cancer survival rates (%) each year after diagnosis by sex [90]. The blue line represents males, whilst the pink line represents females

Figure 4 highlights the complex and dynamic pattern of sex differences in lung cancer over time. Male incidence of diagnosis remains steady (Fig. 4A), and shows decreased mortality rate (Fig. 4B). In comparison, females have demonstrated increasing incidence and mortality rates over the last 40 years. Interestingly, females demonstrate higher survival rates for all histological subtypes of lung cancer [85] after accounting for the stage at diagnosis, age and treatment (represented in Fig. 4C) [76]. Notably, females tend to be diagnosed with lung cancer at a younger age, potentially enabling better opportunities for treatment before disease progression [86]. An Australian longitudinal cohort study by Yu et al. found that although women have higher diagnosis rates, males demonstrate a 43% increased risk of lung cancer mortality [11, 87]. The authors identified female patients were significantly more responsive to treatments, which is supported by multiple other studies potentially contributing to increased survival rates [76, 77], whilst other studies identified sex-specific benefits depending on the treatment [88, 89]. Yu et al. identified an increased density of B-cells in the adenocarcinoma tissues of females, which they suggested might contribute to improved treatment and survival outcomes, as B cells have critical anti-tumour activity. Furthermore, as female patients tend to be younger, they also show better baseline health status compared to male counterparts at a similar stage of disease [87].

The innate and adaptive immune responses between males and females differ significantly. Studies show that males with a suppressed immune response are more susceptible to developing malignancies and infections than females [91]. PD-1, an immune checkpoint inhibitor, is increased in female NSCLC patients compared to males, with higher expression in female CD4 + T cells [92]. This pattern was postulated by Gu et al. to be associated with increasing testosterone levels in females with NSCLC, however, this mechanism requires further exploration [92]. Therefore, a complex relationship between lung cancer and biological sex exists. Although females are diagnosed with lung cancer more often, there is a clear bias towards worse outcomes in males. Furthermore, a distinct histological profile exists between the sexes, with unique pathological profiles potentially apparent between males and females. These differences may contribute to variations in response to antitumor and immunotherapy treatments. The trend of lung cancer appears to be linked to social tobacco smoking patterns. However, there is a distinct shift towards increased non-smoking-related lung cancer development rates. This trend implies important genetic factors may promote epidemiological and pathologic differences between males and females.

### The sex hormones: estrogen and testosterone

Sexual dimorphism is apparent in various diseases, especially in the lung. The most common and easily attributable factor that explains differences between males and females are the sex hormones. These steroid hormones are critical factors driving phenotypic differences between males and females. Androgens (testosterone) are the primary male hormone, and estrogens (estradiol) are the predominant female hormone. However, it should be noted that both androgens and estrogens are found in females and males, but at lower physiological concentrations.

Estrogen and testosterone are commonly thought only to be produced by the sex organs (ovaries and testes). However, their effects extend to peripheral tissues in a paracrine manner [93]. As such, sex steroids are implicated in various diseases impacting multiple organ systems, from cardiovascular to neurological and respiratory [35]. Clinical and epidemiological studies have associated sex hormones in modulating lung diseases. The sex hormones can alter airway tone and modulate the inflammatory response, with estrogen driving a pro-inflammatory environment, whilst androgens are reported to have anti-inflammatory activity [94, 95]. This difference in effect potentially contributes to worse outcomes and increased incidence of complications in females suffering lung diseases associated with inflammation [35].

Elevated testosterone levels are linked with decreased asthma risk regardless of sex [96]. Higher levels of androgen receptor expression in human airway epithelium are associated with better lung function and fewer asthma symptoms [97]. This observation is potentially driven by the ability of androgens to attenuate inflammatory factors in the lung [32, 97] and, by inducing airway smooth muscle relaxation through decreased levels of cellular calcium [98]. Dijkstra et al. identified polymorphisms in estrogen receptor alpha (ERα) that are closely linked with airway hyperresponsiveness and worse lung function decline in females [99]. Even in the absence of stimulation, ERα deficient mice demonstrate significant AHR [100], highlighting a close relationship between estrogen and asthma symptoms.

Generally, estrogens are considered immunological enhancers (i.e. promote an immune response), and androgens/testosterone are immunological suppressors (i.e. reduce the immune response) [101]. It is hypothesised that increased estrogen levels enhance the T2 immune profile in asthma, causing the development and an increased frequency of exacerbations in asthma [101]. This notion is supported by evidence of peri-menstrual worsening of asthma symptoms reported in 20–40% of females with severe or difficult-to-control asthma [102, 103]. Symptoms appear worst during the mid-luteal phase (estrogen and progesterone are elevated) [104]. Many immune cells that predominate in asthma express estrogen and testosterone receptors such as eosinophils, airway smooth muscle cells and T cells [101]. As such, hormonal fluctuations throughout the menstrual cycle influence the immune response to allergic stimuli [105]. Epidemiological evidence shows that the rate of COPD incidence in females is increasing, with the death rate in females increasing since 2000 [106]. Differences in COPD pathology between males and females may be partly modulated by estrogen. Mouse models show female mice develop more airway obstruction upon chronic cigarette smoke exposure, while male mice develop more emphysema. However, ovariectomised female mice (to remove estrogenic effects) develop emphysema similar to male mice [63]. This indicates that estrogen promotes a different pathological COPD phenotype in females, contributing to worse disease outcomes. Androgens have been shown to have anti-inflammatory properties and regulate the structure and function of non-reproductive organs. Further, increased testosterone levels are linked with a decreased risk of asthma in both males and females [96]. Chiarella et al. [107] conducted an extensive review outlining the varying effect estrogen has on multiple airway cell types, highlighting the complex interaction between the sex hormones and the lung.

Androgens have been implicated in lung cancer development, with reduced androgen levels associated with decreased cell proliferation [108] and found to alter the gene expression profile in cancer cell lines [109]. Testosterone is believed to function as a promoter of tumour cell proliferation, contributing to the higher incidence and worse outcomes from cancer in men [110]. However, estrogen has also been linked to the incidence of NSCLC in females—compounding the adverse effects of cigarette smoking. Females who smoke using estrogen replacement therapy (ERT) indicated more than double an increased risk for adenocarcinoma development. Whereas those using ERT and who have never smoked showed no increased odds of developing lung cancer [78].

#### Puberty, pregnancy and menopause

We have discussed how sex-specific patterns of asthma incidence change in puberty. It is important to recognise that both estrogen and testosterone change during puberty and are active in both sexes. The dramatically increased estrogen production in females at puberty potentially promotes increased immune system responsiveness and airway smooth muscle contraction [101, 111]. Conversely, increased testosterone in males is likely protective, suppressing eosinophil and neutrophil inflammation in the lungs and improving airway tone [112]. Further, Bulkhi et al. found a one unit log_2_ increase of serum testosterone was associated with an 11% decreased risk of asthma in males and a 10% decrease in females [96]. However, no correlation between serum testosterone and current asthma was reported for patients under 12 years old. This highlights that childhood asthma is promoted by non-hormonal factors and requires further investigation.

An increasing prevalence of asthma in pregnancy has been reported overtime, from 3.7% in 1997 to 8.4% in 2001 [113], with rates as high as 12.7% in Australia in 2012 [114]. Approximately 20% of females with asthma experience increased exacerbations during pregnancy [115]. The mechanical implications due to uterus enlargement combined with hormonal changes during pregnancy cause increased asthma symptoms in pregnant females [116]. Hormonal changes occur in pregnancy to fulfil the mother's and fetus metabolic needs. As detailed earlier, estrogen and progesterone modulate the immune response, which can lead to worse asthma symptoms. Up to 40% of mothers report that changes to their asthma vary with successive pregnancies, indicating that a complex interplay of factors affects asthma in pregnancy [117].

Pregnancy with concurrent COPD or lung cancer is rare as both conditions develop later in life. Only two instances of pregnancy in patients with COPD have been reported. In one example, COPD symptoms improved with pregnancy, potentially due to the protective role of estrogen against increased bronchoconstriction [118]. The patient's condition significantly declined post-delivery, indicating that the pregnancy caused a partial reversal of COPD progression. In contrast, the other case of COPD in pregnancy [119] indicated little to no improvement, potentially due to the overall worse disease state of the patient. Limited data and studies are evaluating lung cancer's molecular and genomic features in pregnancy. However, adenocarcinoma is the most common form of lung cancer in pregnancy (80%), which may be linked to increased estrogen receptor expression in this cancer type [120, 121]. Consistent with general patterns for cancer, pathological characteristics and health outcomes for patients with lung cancer are the same irrespective of the pregnancy [120, 122].

Early menarche is closely linked with faster lung function decline and worse health outcomes later in life [13], with smoking known to induce early menopause. Menopause is characterised by a distinct reduction of progesterone and estrogen production in females, occurring around the fifth decade [123]. Generally, postmenopausal females have a significantly reduced risk of developing asthma [124]. However, females with asthma at menopause have high levels of circulating estradiol, with a dose-dependent correlation with asthma severity [125]. Asthma prevalence increases in males compared to females after 50 years of age, coincidentally when testosterone levels decrease further [126]. A recent systematic review [123] determined that the contribution of menopause to asthma remains conflicting due to sources of bias and heterogeneity in the current literature. The authors posit that it may be prudent to explore the relationship between menopause and specific asthma phenotypes, which may lead to more insightful conclusions. Only two studies have investigated the link between menopause and COPD, with both finding no association [127, 128]. Hormone replacement therapy (HRT) increased the risk of adult-onset asthma by 49% in menopausal females in two independent cohorts [124, 129]. This highlights a complex interaction between asthma, menopause and hormone changes that requires deeper investigation. Early menopause is linked to an increased risk of lung cancer [130]; although smoking can bring forward the onset of menopause, this may primarily be a smoking effect. Alternatively, some studies have found late menopause (older than 55 years) is linked with an increased risk of lung cancer among non-smokers [131, 132]. This pattern may be caused by greater life-long exposure to estrogen, which has been linked with the development of other cancers [133]. Inconsistent definition of disease outcomes and measurements in studies investigating and associating menopause is a significant limiting factor. As a result, findings from these studies generate conflicting results. The use of clear clinical definitions and the examination of disease subtypes will enable more valuable and insightful conclusions to improve the current understand of the link between the sex hormones with asthma, COPD and lung cancer.

A small cohort study of healthy young females demonstrates no change in multiple lung function measurements across all menstrual cycle stages [134]. Although, a minor positive correlation between tidal volume, inspiratory time and expiratory time was reported with estradiol and progesterone during the early-to-mid luteal phase. A study by Hanley in 1981 measured peak expiratory flow rate (PEFR) in 102 female asthmatic patients [135]. Of the 36 patients who reported worsened symptoms at the start of menstruation, PEFR indicated a significant reduction. This indicates that an increase in airway resistance prompted the perception of worse symptoms. However, 65% of the cohort reported no change in symptoms, highlighting that the effect of menstruation on asthma symptoms is inconsistent. A recent similar study combined subjective and objective measurements of premenstrual asthma deterioration in 103 females with asthma. 60% of participants described worsened symptoms in at least one of two menstrual cycles. However, only three females presented with objective deterioration in peak flow rates [136]. An association between the start of the menstrual cycle and asthma symptoms exists; however, there is a discrepancy between the perception of symptoms and physiological changes. Clearly, a highly complex interaction exists between hormone levels, lung physiology and psychological perception of symptoms. Further investigation of this relationship is necessary to improve patient care and health outcomes and our understanding of disease pathophysiology.

Sex hormones influence the pathophysiology of lung diseases. The exact role and mechanism of how estrogen and testosterone function is yet to be wholly elucidated. The current evidence indicates an association between estrogen and testosterone with clinical symptoms and presentation of these diseases, with no well-defined link to disease development mechanisms between the sexes. There remains a dearth of knowledge surrounding the differential effects of sex hormones in both healthy and disease conditions. Although the role of sex hormones is apparent, a deeper exploration of their signalling and mechanical pathways is required to elucidate how estrogen and testosterone contribute to disease development. The implication of alternate pathogenic factors driving sex differences is evidenced in children where the sex hormonal effect is limited and clear patterns of sex differences exist.

### The sex chromosomes

An imbalance exists in disease susceptibility and severity between males and females, which is apparent pre-puberty [6], removing the effects of sex hormones. As such, this draws attention towards fundamental genetic differences between males and females. The concept of sex-biased gene expression is well-established and reviewed in detail by Grath and Parsch [137]. The processes driving sex-biased expression are complex. In particular, sex-chromosome-specific mechanisms such as dosage compensation directly contribute to sexually dimorphic gene expression. Genes on the X and Y chromosome have been shown to contribute to critical cellular processes and are linked to various diseases [138–141]. Therefore, this is a fundamental difference between males and females which may contribute to sex differences in disease susceptibility, progression and severity.

Human cells contain 23 pairs of chromosomes, with 22 pairs referred to as autosomes and the final pair called the sex chromosomes, X and Y. Females have two X-chromosomes (X-chr), and males have one X-chr and one Y-chromosome (Y-chr). The expression of the *SRY* gene from the Y-chr initiates the development of male genitalia, demonstrated by the seminal 'four-core genome' (FCG) mouse model [142]. This model involved transposing *SRY* from the Y-chr to an autosome, meaning that XX and XY mice with ovaries and XX and XY mice with testis could be bred [142]. As a result, it was possible to distinguish whether differences in gene expression from sex chromosome complement drive a sexually dimorphic phenotype or sex hormones [143]. The FCG model has been applied across a range of experimental designs, which Arnold et al. reviewed in detail and highlighted the importance of this model across different disease systems [143]. In one iteration, distinct differences were observed between XX and XY mice with the same gonadal type, implying a lack of effect by gonadal hormone secretions [144]. Although the effects of estrogen and testosterone must be acknowledged, these models indicate distinct X and Y-chr-specific regulation. In support, studies show that genetic factors drive most differences between the sexes in specific tissues [145, 146].

The sex chromosome complement is a critical biological factor driving sexual dimorphism in disease. The Y-chromosome contains the fewest number of genes (72) compared to the X-chromosome’s 833 genes, highlighting a clear imbalance in genotype between males and females. A study investigated the male-specific region (MSY) of the Y-chromosome, identifying unique haplogroups and observed a 50% increased risk for coronary artery disease compared to the other haplotypes [147]. Macrophages from the males carrying the susceptible haplogroup indicated altered processes of inflammation. A wealth of studies and reviews highlight the contribution of the Y-chromosome in protecting against or increasing susceptibility to various diseases [147–150].

The importance of the Y-chr is controversial. It is accepted that the X and Y-chr were once identical, with evolutionary studies demonstrating that they share a common ancestor chromosome. A divergence event causes the Y-chr to undergo significant changes, which results in the partial degeneration of its structure, with some evidence this degradation is continuing [151]. Studies have forecasted that the Y-chromosome is declining, with a steady loss of genes over millions of years. This begged the question, “How important is the Y-chromosome, and will it disappear?” This is a hotly debated topic with two competing schools of thought:

A) Degradation of the Y-chromosome will continue until it eventually becomes extinct
B) Y-chromosome degradation is slowing down, with the remaining genes being critical for survival

The X-chr undergoes a unique process to adjust for this dramatic degradation of the Y-chr, where one X-chr becomes inactivated. X-chr inactivation (XCi) occurs early in human development. Either the maternal or paternal X-chr becomes inactivated in each cell. The same X-chr remains inactivated throughout the mitotic proliferation of that cell [152]. The non-coding RNA “*Xist*” initiates the recruitment of chromatin-modifier proteins, resulting in transcriptional silencing [153]. The silenced X-chr undergoes structural and epigenetic remodelling leading to the formation of a condensed Barr body. This process theoretically accounts for the double dosage of two X chromosomes in females compared to one in males. However, XCi is incomplete, with several genes escaping the inactivation process [146, 154]. Approximately 15–25% of genes escape XCi [155], meaning that these genes are expressed from both X-chromosomes in females. As such, females experience ‘double-dosage’, whilst males only have a ‘single dose’ of these genes. Variations in XCi have also been implicated in disease susceptibility [154].

**Fig. 5:**
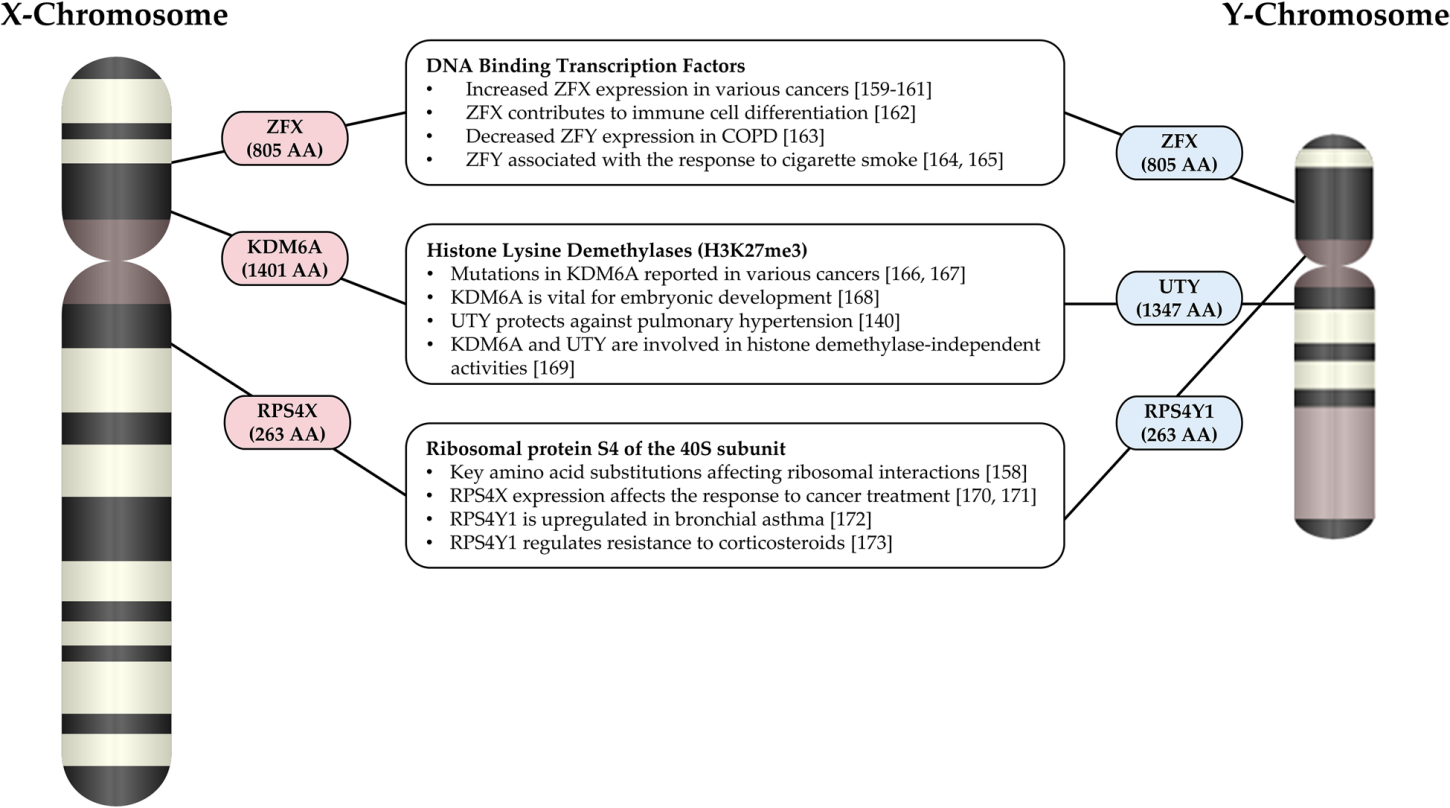
Illustration of genome regulators on the X and Y chromosome and their contribution to various diseases. *AA* amino acids, *H3K27me3* Histone 3 Lysine residue 27 trimethylation [140, 158–171, 171–173]

Bellott et al. [156] compared the Y-chromosomes of multiple mammal species to investigate whether an overlap existed for the evolutionarily conserved genes. The authors identified many conserved genes that carried functions beyond sex determination and function to affect all levels of the central dogma—from gene expression to protein translation. Therefore, genes that have survived on the Y-chromosome are critical regulators of various cellular processes. These genes include *RPS4Y1*, *ZFY*, *DDX3Y*, *EIF1AY*, *KDM5D*, *KDM6C*. These Y-linked genes have X-chromosome counterparts which are also evolutionarily conserved [156]. However, the sequences of these genes are non-exact, resulting in these proteins having structural variances affecting biological systems through divergent mechanisms and pathways [138, 148, 157]. Therefore, these genes represent fundamental sexual dimorphism at a genetic level, which exists in every cell type. As these genes contribute to the regulation of normal cell and molecular processes at a whole genome level [158], an imbalance of function may contribute to sex differences in disease susceptibility, progression and the response to clinical interventions. The function of some of the sex chromosome-linked homolog genes and their differences are presented in Fig. 5.

## Conclusions

Sexual dimorphism is prevalent in various diseases and particularly complex in respiratory diseases—asthma, COPD and lung cancer. These differences affect the susceptibility, severity, presentation and response to medical treatments. Despite the increased study of the factors contributing to sex differences in recent years, more research is required. The sex hormones estrogen and testosterone are well-recognised to contribute to the severity of disease, but how and whether they are the primary factors causing disease pathogenesis remains unclear. We have described the phenomena of dosage compensation and XCi escape causing an imbalance of key genome regulators from the sex chromosomes. These genes contribute to central disease features such as inflammation and fibrosis. Therefore, they are valuable candidates to further our understanding of the development of disease and the generation of new clinical interventions to improve the health outcomes for males and females.

## Data Availability

Not applicable.

## Abbreviations

COPD: chronic obstructive pulmonary disease
FEV_1_: forced expiratory volume in one second
ICS: inhaled corticosteroid
TNFα: tumour necrosis factor alpha
ECM: extracellular matrix
FAO: fixed airflow obstruction
AHR: airway hyperresponsiveness
TGFβ: transformation growth factor beta
SEO-COPD: Severe early onset chronic obstructive pulmonary disease
NSCLC: non-small cell lung cancer
SCLC: small cell lung cancer
ERT: estrogen replacement therapy
HRT: hormone replacement therapy
PEFR: peak expiratory flow rate
X-chr: X chromosome
Y-chr: Y chromosome
FCG: four core genome
XCi: X chromosome inactivation
